# Evaluating the Effectiveness, Tolerability, and Safety of Eptinezumab in High-Frequency and Chronic Migraine in Real World: EMBRACE—The First Italian Multicenter, Prospective, Real-Life Study

**DOI:** 10.3390/brainsci14070672

**Published:** 2024-06-30

**Authors:** Piero Barbanti, Bianca Orlando, Gabriella Egeo, Florindo d’Onofrio, Alberto Doretti, Stefano Messina, Massimo Autunno, Roberta Messina, Massimo Filippi, Giulia Fiorentini, Cristina Rotondi, Stefano Bonassi, Cinzia Aurilia

**Affiliations:** 1 Headache and Pain Unit, IRCCS San Raffaele, Via della Pisana 235, 00163 Rome, Italygabriella.egeo@sanraffaele.it (G.E.);; 2 San Raffaele University, 00166 Rome, Italy; 3 Headache Center Neurology Unit, San Giuseppe Moscati Hospital, 83100 Avellino, Italy; 4 Laboratory of Neurosciences, Department of Neurology-Stroke Unit, Istituto Auxologico Italiano, IRCCS, 20145 Milan, Italy; 5 Department of Clinical and Experimental Medicine, University of Messina, 98122 Messina, Italy; mautunno@unime.it; 6 Neurology Unit, IRCCS San Raffaele Scientific Institute, Vita-Salute San Raffaele University, 20132 Milan, Italy; 7 Clinical and Molecular Epidemiology, IRCCS San Raffaele, 00166 Rome, Italy

**Keywords:** migraine, eptinezumab, anti-CGRP mAbs, treatment, real-life, disability

## Abstract

We conducted a multicenter, prospective study (EMBRACE) evaluating the real-life effectiveness, safety, and tolerability of eptinezumab (100 mg/300 mg)—a monoclonal antibody targeting the calcitonin-gene-related peptide (anti-CGRP mAb)—in high-frequency episodic migraine (HFEM) or chronic migraine (CM). The primary endpoint was the change in monthly migraine days (MMD) for HFEM or monthly headache days (MHD) for CM at weeks 9–12 compared to baseline. The secondary endpoints included changes in monthly analgesic intake (MAI), Numerical Rating Scale (NRS), Headache Impact Test (HIT-6), Migraine Disability Assessment Scale (MIDAS), Migraine Interictal Burden Scale (MIBS-4), and responder rates. The safety analysis involved 44 subjects; the effectiveness analysis included 26 individuals. Eptinezumab was well-tolerated. In CM patients, eptinezumab significantly reduced MHD (−16.1 ± 9.9, *p* < 0.001), MAI, NRS, HIT-6, MIDAS, and MIBS-4. In HFEM patients, it significantly reduced NRS, HIT-6, MIDAS, and MIBS-4, though reductions in MMD (−3.3 ± 4.5) and MAI were not statistically significant. Overall, ≥50% and ≥75% response rates were 61.5% and 30.8%, respectively (60% and 30% in non-responders to subcutaneous anti-CGRP mAbs). The clinical change was rated as much or very much improved by 61.0% of the patients. Eptinezumab demonstrated high effectiveness, safety, and tolerability in real-life among hard-to-treat migraine patients with multiple treatment failures, including anti-CGRP mAbs.

## 1. Introduction

Migraine prevention should be considered for patients experiencing at least two disabling migraine days per month, even with optimized acute treatment, to mitigate the risk of disease progression [1,2]. Despite this recommendation, the proportion of patients taking migraine preventive medication remains low, largely due to their overall low tolerability and inadequate disease awareness among patients. The recent availability of drugs targeting the calcitonin-gene-related peptide (CGRP), known for their favorable efficacy-to-tolerability ratio, holds the potential to facilitate a larger proportion of migraine sufferers in receiving appropriate prophylactic therapy [3].

Eptinezumab, a humanized monoclonal antibody (mAb) targeting the CGRP, is indicated for migraine prophylaxis and is distinguished by the intravenous (iv) administration route [4]. Its linear pharmacokinetics result in peak concentration (Cmax) attainment at the end of iv administration, with values of 37.3 μg mL^−1^ for a single 100 mg dose and 114 μg mL^−1^ for a single 300 mg dose. Notably, the time to maximum plasma concentration (Tmax: 30 min for a 30-min iv administration) is significantly shorter when compared to subcutaneous anti-CGRP mAbs such as erenumab (4–11 days), fremanezumab (5–11 days), and galcanezumab (7–14 days) [5]. This unique pharmacokinetic profile accounts for its exceptionally rapid onset of action, as evidenced by the ability to shorten the time to headache and symptom resolution compared to placebo in moderate to severe migraine attacks [6]. With a terminal half-life of 27 days and modest exposure metrics required to reach concentrations achieving 90% of the maximum change in effect (EC_90_), eptinezumab is suitable for quarterly administration at doses of both 100 mg and 300 mg [5].

The efficacy of eptinezumab has been documented in patients with episodic migraine (EM) or chronic migraine (CM), even in presence of multiple therapeutic failures [4,7,8,9,10]. In 12-week randomized controlled trials (RCTs), eptinezumab effectively reduced monthly migraine days (MMD) in EM of 3.9 days at 100 mg (*p* = 0.0182) and 4.3 days at 300 mg compared to a reduction of −3.2 days in the placebo group (*p* = 0.0001) [8]. In CM, it decreased monthly headache days (MHD) with placebo showing a reduction of 5.6 days, 100 mg demonstrating a reduction of 7.7 days (*p* < 0.0001), and 300 mg resulting in a reduction of 8.2 days (*p* < 0.0001) [9]. In migraine patients with two-to-four previous preventive treatment failures, eptinezumab was also significantly more effective than placebo in reducing MMD from baseline to weeks 1–12 (100 mg: −4.8; 300 mg: −5.3; placebo: −2.1; *p* < 0.0001) [10]. In all RCTs, eptinezumab exhibited a favorable safety and tolerability profile [7].

Given its optimal efficacy–tolerability ratio and remarkable speed of action, eptinezumab may be especially beneficial for hard-to-treat migraine patients, particularly those characterized by CM, medication overuse, and prior therapeutic failures. These challenges are frequently encountered in daily clinical practice at headache centers. However, RCT data must be reproduced in real-life studies. The former are conducted in highly controlled settings with carefully selected participants who often have few or no additional medical conditions. Consequently, RCTs determine the efficacy, safety, and tolerability of a drug in a group that may not accurately reflect the broader, more diverse population. Conversely, real-life data are typically gathered from subjects with complex clinical scenarios, characterized by more severe forms of the disease, comorbidities, and concomitant medications. Real-life studies are invaluable for assessing treatment effectiveness, shedding light on the long-term effects of treatment, and helping identify potential predictors of response. Most notably, real-life investigations are crucial for assessing drug tolerability and safety, as they include older patients and those with comorbid conditions.

Subcutaneous monoclonal antibodies have proven to be even more effective in multicenter, prospective, real-world studies compared to what was documented in clinical trials. Therefore, there is anticipation to ascertain the effectiveness of eptinezumab in the real-life population [11,12,13].

To address this, we set up an Italian multicenter, prospective, cohort, real-life study named EMBRACE (eptinezumab in real life in Italy). The study focuses on adults with high-frequency episodic migraine (HFEM) or chronic migraine (CM) treated with eptinezumab who have previously experienced multiple therapeutic failures. The EMBRACE study started in February 2023, includes multiple headache centers across different Italian regions, and is ongoing.

In the present paper, we present the 12-week effectiveness, safety, and tolerability data from the EMBRACE study.

## 2. Methods

EMBRACE, registered with ClinicalTrials.gov ID: NCT05570149, is an ongoing Italian multicenter, prospective, real-life study [14] that began on 24 February 2023 with the last data analysis performed on 20 May 2024. The study serves as a supplementary investigation within the framework of the Italian Migraine Registry (I-GRAINE). We included all consecutive adult patients affected by HFEM (≥8 migraine days/month) or CM (≥15 headache days/month) who were treated with intravenous eptinezumab, either 100 mg or 300 mg quarterly, in five headache centers across four Italian regions (Lombardy, Latium, Campania, and Sicily). The initial eptinezumab dose, whether 100 mg or 300 mg, was selected based on each patient’s preference. Eptinezumab was diluted in 100 cc of 0.9% saline solution and administered intravenously over a period of 30 min. Patients were under observation for a minimum of 30 min following the conclusion of each infusion.

We excluded patients with a history of clinically significant cardiovascular or cerebrovascular disease.

All subjects underwent a thorough physical and neurological examination conducted by specifically trained, board-certified neurologists. The patients’ information was gathered with face-to-face interviews using a shared, semi-structured, questionnaire carefully detailing socio-demographic features (age, sex, body mass index), migraine characteristics (age at migraine onset; migraine type and frequency; pain side, quality, and severity; accompanying symptoms; unilateral cranial autonomic symptoms and cranial autonomic parasympathetic symptom scale; dopaminergic symptoms; allodynia and allodynia symptom checklist-12; headache impact test; migraine disability assessment scale, migraine interictal burden scale-4), migraine treatments (monthly analgesic intake; number, type and responsiveness/failure to prior prophylaxis; concomitant prophylaxis; botulinum toxin responsiveness; triptan responsiveness), comorbidities (psychiatric, cardiovascular, gastro-intestinal, endocrinological, gynecological, respiratory, immunologic, diabetes, oncological, other) and concomitant medications (table).

During a 28-day preliminary phase and throughout the study duration, the participants were asked to fill a paper–pencil diary to monitor MMD (for patients with EM), MHD (for those with CM), monthly analgesic intake (MAI), and pain intensity (using the Numerical Rating Scale score, NRS). Migraine disability was assessed using the Headache Impact Test (HIT-6) and the Migraine Disability Assessment Scale (MIDAS), while interictal migraine burden was evaluated using the Migraine Interictal Burden Scale (MIBS-4). atients’ satisfaction with eptinezumab was evaluated using the Patient Global Impression of Change (PGIC). Additionally, individuals were encouraged to promptly report any adverse events experienced during the study period. 

Regarding the definition of MMD, we followed the standardized definition proposed by van der Arent et al., which considers a migraine day to be (1) a day with a headache lasting at least 30 min, meeting the criteria for migraine without aura as defined by the B and C criteria of the International Classification of Headache Disorders, 3rd edition, or (2) a day with a visual aura lasting between 5 to 60 min, or a day with a headache for which acute migraine-specific medication is used, regardless of its effectiveness [15,16].

In patients with CM, we opted to assess MHD, a measure that includes any headache day, encompassing both migraine-like and tension-type headache days. This decision was made because differentiating headache days from migraine days in a real-life CM population and setting may be challenging (a tension-type like headache may precede the development of a full-blown migraine attack: in this case, an early and effective treatment with unspecific acute migraine drugs might lead the patient to classify a migraine day as a headache day).

The primary endpoint was the change in MMD for individuals with HFEM and of MHD for those with CM during weeks 9–12 compared to baseline. Secondary endpoints included changes in MAI, NRS, MIDAS, HIT-6, and MIBS-4 scores, as well as responder rates (≥50%, ≥75%, and 100%) at the same time point compared to baseline. Additionally, the number of migraine days (for HFEM) and headache days (for CM) during the first week following the first eptinezumab administration and PGIC at week 12 were also assessed. The safety and tolerability were evaluated by examining the incidence of adverse events, serious adverse events, and adverse events that resulted in study discontinuation.

Given the exploratory nature of the study, convenience sampling was chosen to facilitate quick data collection and provide initial insights into the RWE concerning the use of eptinezumab in patients with HFEM and CM. Additionally, the sample size of the present study aligns with those used in similar studies.

All participants provided written informed consent before participating in the study. The Institutional Review Board at IRCCS San Raffaele, Roma (RP 19/26) granted approval.

## 3. Statistics

The demographic and clinical characteristics of patients at the time of first visit were expressed as mean and standard deviation (SD) for continuous variables and as proportions for categorical variables. Distribution frequencies were estimated for qualitative variables, and scores for the different scales are reported as mean with their SD. The difference in demographic and clinical selected variables between HFEM and CM patients was tested with the unpaired Student’s *t*-test or the Chi square test. The assumption of normality was assessed by the one-sample Kolmogorov–Smirnov test. Whenever the assumption was not satisfied, the non-parametric Mann–Whitney U test was used. Fisher’s exact test was applied for expected frequencies below 5. The comparison before–after treatment for primary and secondary endpoints was performed with the paired samples Student’s *t*-test (the non-parametric Wilcoxon signed-rank test was used for those variables whose distribution of the differences between the paired observations was not normal). Given the exploratory nature of the study, no adjustment for multiple comparison was applied. All the data were analyzed with the software SPSS v28 for Windows (IBM Corp, .1 New Orchard Road, Armonk, NY 10504-1722, USA).

## 4. Results

**Figure 1:**
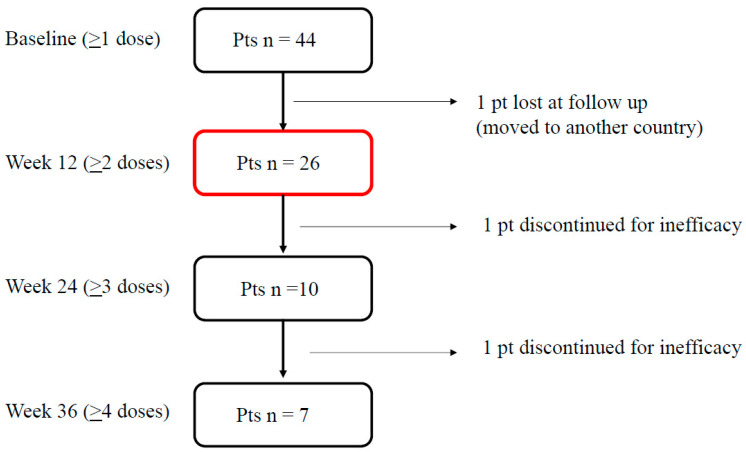
Patients’ disposition.

As of 30 April 2024, a total of 44 subjects with migraine were treated with ≥1 eptinezumab dose and were considered for safety analysis (see Table 1). The efficacy analysis was conducted on the 26 patients who completed the 12-week follow-up. One patient was lost to follow-up at week 12 due to relocating to another country (Figure 1). The characteristics of the patients included in the study are reported in Table 2. All patients experienced an average of 4.4 prior therapeutic failures, including the use of subcutaneous anti-CGRP mAbs in 37% of cases, and exhibited a very high level of migraine disability, as indicated by their HIT-6, MIDAS, and MIBS-4 scores. The first eptinezumab dose was 100 mg in 26 individuals and 300 mg in 1 individual. The patients with CM had higher MIDAS scores (*p* = 0.044) compared to those with HFEM.

**Figure 2:**
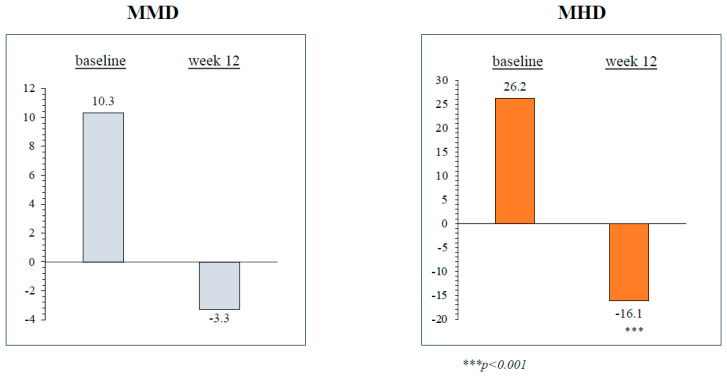
Change in monthly migraine days (MMD) in patients with high-frequency episodic migraine (HFEM) and in monthly headache days (MHD) in patients with chronic migraine (CM) from baseline to weeks 9–12.

**Figure 3:**
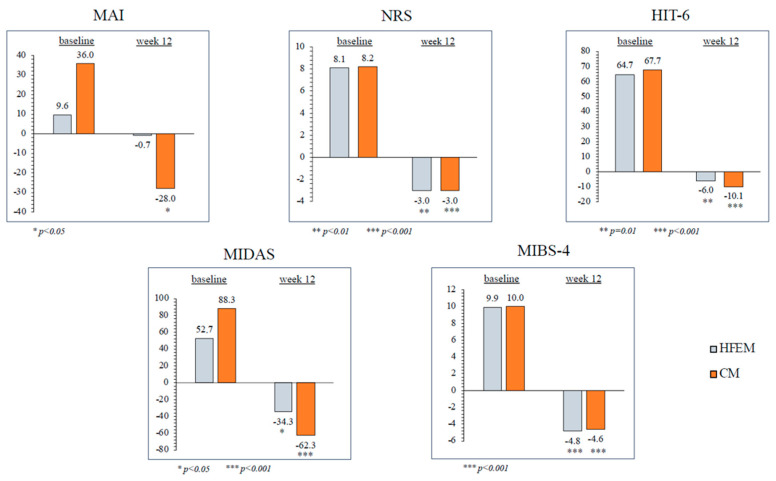
Change in monthly analgesic intake (MAI), Numerical Rating Scale (NRS) score, Headache Impact Test-6 (HIT-6) score, Migraine Disability Assessment Scale (MIDAS) score and Migraine Interictal Burden Scale (MIBS-4) score from baseline to weeks 9–12 in patients with high-frequency episodic migraine (HFEM) and in those with chronic migraine (CM).

At weeks 9–12, eptinezumab significantly reduced MHD in subjects with CM (−16.1 ± 9.9, *p* < 0.001) compared to baseline. In individuals with HFEM, there was also a decrease in MMD (−3.3 ± 4.5), though this outcome did not achieve statistical significance. Likewise, eptinezumab treatment led to a notable reduction in MAI in CM (−28 ± 44.9, *p* = 0.014), whereas the reduction observed in HFEM (−0.7 ± 5.7) did not reach statistical significance. During the same time interval, eptinezumab induced a statistically significant reduction in NRS (−3 ± 1.7, *p* = 0.004), HIT-6 (−6 ± 4.7, *p* = 0.014), MIDAS (−34.3 ± 28.1, *p* = 0.018), and MIBS-4 (−4.8 ± 1.3, *p* < 0.001) scores in subjects with HFEM, as well as in those with CM (−3 ± 1.9, *p* < 0.001; −10.1 ± 7.6, *p* < 0.001; −62.3 ± 40.5, *p* < 0.001; −4.6 ± 2.6, *p* < 0.001, respectively) (Figure 2 and Figure 3; Table 3).

**Figure 4:**
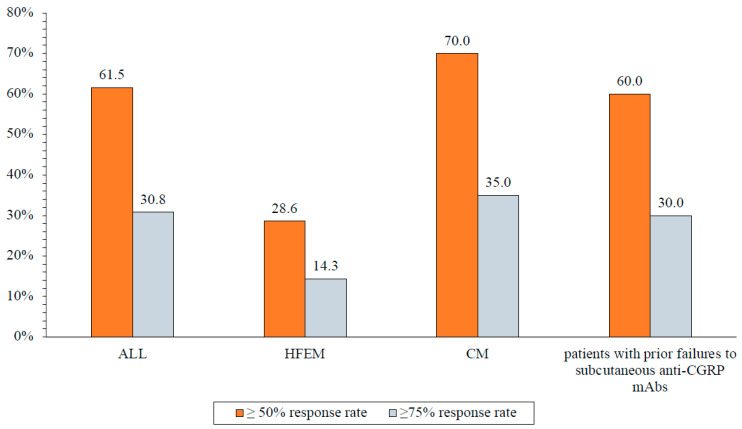
Proportion of subjects with a ≥50% or ≥75% reduction in monthly migraine/headache days at week 12 compared to baseline. All, all patients; HFEM, high-frequency episodic migraine; CM, chronic migraine.

**Figure 5:**
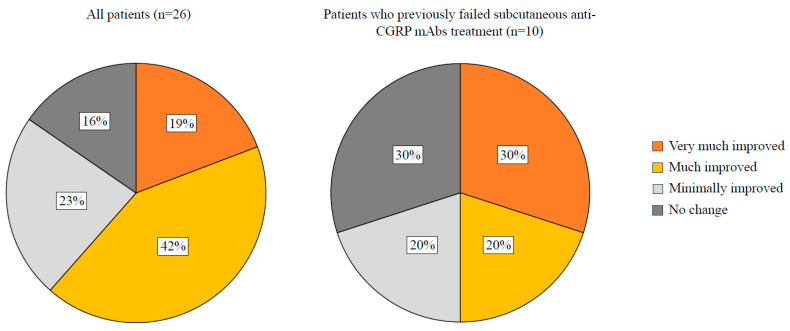
Patients’ assessment of their overall health status at week 12 relative to baseline, utilizing the Patient Global Impression of Change (PGI-C) scale.

The ≥50% and ≥75% response rates were 61.5% and 30.8%, respectively, while no patient achieved a 100% response rate. Specifically, for HFEM, the ≥50% and ≥75% response rates were 28.6% and 14.3%, while for CM, the rates were 70.0% and 35.0% (Figure 4). Among the 10 patients who did not previously respond to ≥1 subcutaneous anti-CGRP mAb, 60.0% showed a ≥50% response to eptinezumab, and 30.0% exhibited a ≥75% response (Figure 4; Table 4 and Table 5). The mean PGIC score at week 12 was 2.1 ± 0.8 (HFEM: 2.3 ± 0.7; CM: 2.1 ± 0.8). The clinical change was rated as much or very much improved by 61.0% of the patients and by 50.0% of those who previously failed subcutaneous anti-CGRP mAbs treatment (Figure 5).

Two out of twenty-seven patients (7.4%) reported immediate headache relief during the first eptinezumab infusion. During the 7 days following the first eptinezumab administration, patients with HFEM experienced an average of 1 ± 1.3 migraine days, while individuals with CM reported 1.1 ± 1.9 headache days.

By week 12, eptinezumab led to a reversion from CM to EM in 80% (16/20) of the patients and from medication overuse to no medication overuse in 76.5% (13/17) of the individuals. No patient reported any adverse events during eptinezumab infusion. One patient (2.3%) reported mild constipation and hair loss during the first trimester of treatment. No serious adverse events were reported, and no patients discontinued treatment due to tolerability issues.

## 5. Discussion

EMBRACE is a prospective, multicenter, ongoing study exploring the effectiveness, tolerability, and safety of eptinezumab in subjects affected by HFEM or CM in real life. In this first report, we document that eptinezumab is highly effective, safe, and well-tolerated among hard-to-treat migraine patients with multiple treatment failures, including anti-CGRP mAbs, frequent comorbidities, and medication overuse.

Eptinezumab—administered at the dose of 100 mg in all but one patient—resulted in significant reductions after 12 weeks in MHD, analgesic consumption, pain severity, migraine disability, and interictal migraine burden, along with an improvement in PGIC to much better or very much better (Figure 5). The reduction in MMD and MAI did not reach statistical significance in individuals with HFEM, likely due to their small number (*n* = 7). Eptinezumab demonstrated optimal tolerability, with no adverse events emerging during infusion and only a single patient (2.3%) reporting mild and transient adverse effects. Notably, despite their clinical complexity, almost 80% of patients experienced remission to the episodic form of migraine and cessation of medication overuse after a single eptinezumab dose. It is worth mentioning that patients who had previously shown no response to subcutaneous anti-CGRP mAbs experienced a significant reduction not only in headache days but also in pain intensity and ictal and interictal disability with eptinezumab (Table 5). They rated the clinical change as much or very much improved in 50% of the cases (Figure 5).

The occurrence of immediate headache relief during the first infusion occurred in 2 patients (7.4%), which aligns with findings from the RELIEF study, which suggest an “added benefit of alleviating an active migraine attack” in patients taking eptinezumab for migraine prevention [6]. Additionally, the low average migraine frequency documented in the present study during the 7 days following the first eptinezumab dose (HFEM: 1 ± 1.3; CM: 1.1 ± 1.9) fits well with the rapid onset of action of the drug, characterized by an advantageous short Tmax [5].

The effectiveness of eptinezumab in the initial EMBRACE report appears to surpass the efficacy reported in RCTs, as previously noted also with subcutaneous anti-CGRP mAbs [11,12,13]. One possible explanation for this phenomenon is a putative increase in CGRP activity in more complex and multifaceted real-life patients, which could emphasize the anti-CGRP therapeutic properties of the treatment [13].

We observed a greater reduction in migraine days (−3.3 and −16.1 compared to −2.7 and −3.2) and a higher number of ≥50% responders (61.5% vs. 42.0% and 49.0%) and of ≥75% responders (30.8% vs. 16.0% and 19.0%) compared to the DELIVER trial, which was conducted in patients with two-to-four previous preventive treatment failures [10]. This finding is particularly compelling when considering that 37.5% of the EMBRACE population had previously not responded to at least 1 subcutaneous anti-CGRP mAb, showing indeed good responsiveness to eptinezumab (≥50% response rate: 60%; ≥30% response rate: 60%). This suggests that eptinezumab’s rapid bioavailability at the trigeminal level may both decrease the latency to benefit and increase the proportion of patients who respond to anti-CGRP therapy.

The main limitation of our work is the small sample of patients included in this first report of the ongoing EMBRACE study. As only one patient used eptinezumab 300 mg as starting dose, our results primarily pertain to the 100 mg dose. Furthermore, our patients were predominantly affected by CM, and among individuals with EM, we focused only on those with HFEM, not considering those with lower migraine frequency. The small number of patients with HFEM is likely responsible for the lack of significance in the reduction of MMD and MAI. It is, therefore, plausible that as recruitment continues, a larger number of HFEM patients will lead to statistically significant outcomes for these efficacy endpoints. Lastly, another limitation is the use of paper-and-pencil diaries instead of electronic ones. The dataset’s origin from a nationwide pathology registry ensures its validity. While convenience sampling poses a risk of bias, consecutive patient recruitment, multicenter sourcing, and low withdrawal rates contribute to a low overall risk of bias.

The strengths of our study include its multicenter, prospective design, which enhances the generalizability of our findings. Additionally, the use of a shared, detailed questionnaire for collecting comprehensive demographic and clinical patient features ensures standardized data collection across multiple sites. We also focused on interictal disability to better evaluate the impact of eptinezumab on the daily lives of migraine patients. Furthermore, the inclusion of patient-reported outcomes, such as the PGIC, among the endpoints allows for a more comprehensive assessment of treatment effects, capturing patient perspectives on improvement beyond clinical measures.

## 6. Conclusions

This report highlights the excellent effectiveness and tolerability ratio of eptinezumab in subjects with extremely disabling migraine, often affected by severe medication overuse, unresponsiveness to multiple preventive medications, including subcutaneous anti-CGRP mAbs, and multiple comorbidities. The EMBRACE trial is expected to validate these findings in a larger patient cohort and provide valuable insights into the ongoing debate regarding the potential effectiveness of eptinezumab in patients who are non-responsive to subcutaneous anti-CGRP mAbs. Eptinezumab’s very rapid onset of action could be particularly beneficial for patients with medication overuse, as it may help address the challenge of withdrawing from analgesics. This issue is a significant clinical challenge for some migraine patients, and the prompt effectiveness of eptinezumab may facilitate a smoother transition away from analgesic overuse, potentially leading to improved outcomes and better management of medication-related complications. Additionally, the EMBRACE trial aims to contribute to the individualization of optimal eptinezumab dosages for different migraine patient populations. Although RCTs have shown no significant difference in efficacy and tolerability between the 100 mg and 300 mg eptinezumab doses, it is important to identify which patients might benefit from starting with the 300 mg dose or when it might be appropriate to escalate from 100 mg to 300 mg in real-world settings. This is particularly relevant for subjects with complex clinical profiles or those who have an unsatisfactory response to the initial treatment.

## Figures and Tables

**Table 1:** Demographic and clinical features of patients with high-frequency episodic migraine (HFEM) or chronic migraine (CM) treated with at least one eptinezumab dose.

	All Patients	HFEM	CM
**Patients**	44	7	37
**Age**, *years*	49.7 ± 11.3	50.7 ± 14.2	49.5 ± 10.9
**Females**	36 (81.8%)	6 (85.7%)	30 (81.1%)
**BMI**	23.4 ± 2.7	23.7 ± 3.6	23.4 ± 2.6
**Age at onset**, *years*	17.6 ± 7.6	17.6 ± 10.7	18.2 ± 9.1
**MMD**	10.3 ± 2.7	10.3 ± 2.7	-
**MHD**	26.5 ± 4.9	-	26.5 ± 4.9
**NRS score**	8.1 ± 1.1	8.1 ± 0.7	8.1 ± 1
**MO**	29 (65.9%)	-	29 (78.4%)
**MO duration**, *years*	7.6 ± 4.9	-	7.6 ± 4.9
**Unilateral pain**	31 (70.4%)	4 (57.1%)	27 (73.0%)
**Unilateral cranial autonomic symptoms**	16 (36.4%)	3 (42.8%)	13 (35.1%)
**CAPS score**	0.9 ± 1.7	1.4 ± 1.5	0.9 ± 1.6
**Allodynia**	10 (22.7%)	1 (14.3%)	9 (24.3%)
**ASC-12 score**	1.8 ± 3.5	0.3 ± 0.8	1.5 ± 3.2
*0–2*	36 (81.9%)	7 (100%)	29 (78.4%)
*3–5*	3 (3.7%)	-	3 (8.1%)
*6–8*	5 (7.4%)	-	5 (13.5%)
**Dopaminergic symptoms**	15 (34.1%)	1 (14.3%)	14 (37.8%)
**Monthly analgesic intake**	25.3 ± 17.7	9.6 ± 4.0	26.9 ± 17.5
**Triptan responders** *	24 (80%)	4 (55.2%)	20 (80.0%)
**Onabotulinum toxin A responders** **	10 (58.8%)	2 (28.2%)	8 (40.0%)
**Pts using concomitant prophylaxis**	7 (15.9%)	1 (14.3%)	6 (16.2%)
**Prior treatment failures**	4.9 ± 2.5	3.9 ± 2.3	4.9 ± 2.5
*2–4*	24 (54.5%)	5 (71.4%)	19 (51.4%)
*>4*	18 (40.9%)	2 (28.6%)	16 (43.2%)
**Prior treatment failures with anti-CGRP mAbs** *Withdrawal for inefficacy*	21 (47.7%) 21 (47.7%)	3 (42.9%) 3 (42.9%)	18 (48.6%) 18 (48.6%)
**Pts with ≥1 comorbidity**	25 (56.8%)	3 (42.9%)	22 (59.4%)
**Pts with psychiatric comorbidities**	9 (20.4%)	-	9 (24.3%)
**HIT-6 score**	65.8 ± 5.3	64.7 ± 4.4	65.6 ± 5.5
**MIDAS score**	83.1 ± 46.4	52.7 ± 26.1	86.2 ± 47.2
**MIBS-4 score**	9.8 ± 3.2	9.9 ± 2.1	9.8 ± 3.4
**Eptinezumab dose**			
*100 mg*	43 (97.7%)	7 (100%)	36 (97.3%)
*300 mg*	1 (2.3%)	-	1 (2.7%)

Data are reported as or mean ± SD or proportion (%). HFEM: High frequency episodic migraine, CM: Chronic migraine, BMI: Body mass index, MMD: Monthly migraine days, MHD: Monthly headache days, NRS: Numerical rating scale, MO: Medication overuse, CAPS: cranial autonomic parasympathetic symptoms scale, ASC-12: allodynia symptom checklist, HIT-6: Headache Impact Test-6, MIDAS: Migraine Disability Assessment Scale, MIBS-4: Migraine Interictal Burden Scale. * Proportion calculated on the subjects who were treated with triptans, ** Proportion calculated on the subjects who were treated with onabotulinum toxin A.

**Table 2:** Demographic and clinical features of patients with high-frequency episodic migraine (HFEM) or chronic migraine (CM) completing 12-week follow-up.

	All Patients	HFEM	CM	*p* Value
**Patients**	27	7	20	
**Age,** *years*	47.4 ± 7.3	50.7 ± 14.2	46.2 ± 8.7	0.327
**Females**	21 (77.8%)	6 (85.7%)	15 (75%)	0.557
**BMI**	23.9 ± 3.2	23.7 ± 3.6	23.1 ± 2.2	0.172
**Age at onset,** *years*	18.5 ± 10.5	17.6 ± 10.7	18.8 ± 10.7	0.734
**MMD**	10.3 ± 2.7	10.3 ± 2.7	-	-
**MHD**	26.2 ± 5.0	-	26.2 ± 5.0	-
**NRS score**	8.2 ± 0.8	8.1 ± 0.7	8.3 ± 0.9	0.300
**MO**	17 (63%)	-	17 (85%)	-
**MO duration,** *years*	8.6 ± 5.7	-	8.6 ± 5.7	-
**Unilateral pain**	18 (66.7%)	4 (57.1%)	14 (70.0%)	0.534
**Unilateral cranial autonomic symptoms**	12 (44.4%)	3 (42.8%)	9 (45.0%)	0.636
**CAPS score**	1.4 ± 1.8	1.4 ± 1.5	1.4 ± 1.9	0.428
**Allodynia**	6 (22.2%)	1 (14.3%)	5 (25.0%)	0.400
**ASC-12 score**	0.7 ± 2	0.3 ± 0.8	0.9 ± 2.2	0.154
*0–2*	24 (88.9%)	7 (100%)	17 (85.0%)	0.601
*3–5*	1 (3.7%)	-	1 (5.0%)	
*6–8*	2 (7.4%)	-	2 (10.0%)	
**Dopaminergic symptoms**	13 (48.1%)	1 (14.3%)	12 (60.0%)	0.077
**Monthly analgesic intake**	29.1 ± 38.7	9.6 ± 4.0	36.0 ± 43.1	0.122
Triptan responders *	16 (72.7%)	4 (55.2%)	12 (63.2%)	0.369
Onabotulinum toxin A responders **	8 (30.8%)	2 (28.2%)	6 (31.6%)	0.639
**Pts using concomitant prophylaxis**	5 (18.5%)	1 (14.3%)	4 (20.0%)	0.112
**Prior treatment failures**	4.4 ± 2.3	3.9 ± 2.3	4.7 ± 2.3	0.435
*2* *–4*	17 (63.0%)	5 (71.4%)	12 (60.0%)	0.290
*>4*	10 (37.0%)	2 (28.6%)	8 (40.0%)	0.291
**Prior treatment failures with anti-CGRP mAbs**	10 (37.0%)	3 (42.9%)	7 (35.0%)	0.711 0.711
*Withdrawal for inefficacy*	10 (37.0%)	3 (42.9%)	7 (35.0%)	
**Pts with ≥1 comorbidity**	10 (37.0%)	3 (42.9%)	8 (40.0%)	0. 089
**Pts with** **psychiatric comorbidities**	3 (11.1%)	-	3 (15.0%)	0.390
**HIT-6 score**	67 ± 4.3	64.7 ± 4.4	67.8 ± 4.1	0.107
**MIDAS score**	79 ± 40.6	52.7 ± 26.1	88.3 ± 41.1	0.044
**MIBS-4 score**	10 ± 2.6	9.9 ± 2.1	10.0 ± 2.8	0.903
**Eptinezumab dose**				
*100 mg*	26 (96.3%)	7 (100%)	19 (95.0%)	
*300 mg*	1 (3.7%)	-	1 (5.0%)	

Data are reported as mean ± SD or proportion (%). HFEM: High frequency episodic migraine, CM: Chronic migraine, BMI: Body mass index, MMD: Monthly migraine days, MHD: Monthly headache days, NRS: Numerical Rating Scale, MO: Medication overuse, CAPS: cranial autonomic parasympathetic symptoms scale, ASC-12: Allodynia symptom checklist, HIT-6: Headache Impact Test-6, MIDAS: Migraine Disability Assessment Scale, MIBS-4: Migraine Interictal Burden Scale. * Proportion calculated on the subjects who were treated with triptans, ** Proportion calculated on the subjects who were treated with onabotulinum toxin A.

**Table 3:** Change in monthly migraine days (MMD), monthly headache days (MHD), monthly analgesic intake (MAI), Numerical Rating Scale (NRS) score, Headache Impact Test-6 (HIT-6) score, Migraine Disability Assessment Scale (MIDAS) score and Migraine Interictal Burden Scale (MIBS-4) score from baseline to weeks 9–12 in the whole migraine population (All patients), in patients with high-frequency episodic migraine (HFEM) and in those with chronic migraine (CM).

	All Patients				HFEM				CM			
	Baseline	Weeks 9–12	∆	*p*	Baseline	Weeks 9–12	∆	*p*	Baseline	Weeks 9–12	∆	*p*
Primary endpoint												
MMD	10.3 ± 2.7	7 ± 5.6	−3.3 ± 4.5	*ns*	10.3 ± 2.7	7.0 ± 5.6	−3.3 ± 4.5	*ns*	-	-	-	
MHD	26.2 ± 5	9.8 ± 6.9	−16.1 ± 9.9	*<0.001*	-	-	-		26.2 ± 5	9.8 ± 6.9	−16.1 ± 9.9	*<0.001*
Secondary endpoints												
MAI	29.1 ± 38.8	9.6 ± 7.6	−20 ± 40.1	*0.017*	9.6 ± 4	8.8 ± 5.7	−0.7 ± 5.7	*ns*	36 ± 43	9.2 ± 7.5	−28 ± 44.9	*0.014*
NRS	8.2 ± 0.8	5.3 ± 1.9	−2.9 ± 1.9	*<0.001*	8.1 ± 0.7	5.1 ± 1.7	−3.0 ± 1.7	*0.004*	8.2 ± 0.9	5.1 ± 1.9	−3 ± 1.9	*<0.001*
HIT-6	67 ± 4.3	57.8 ± 7.7	−9 ± 6.9	*<0.001*	64.7 ± 4.4	58.7 ± 7.2	−6.0 ± 4.7	*0.014*	67.7 ± 4	57 ± 7.7	−10.1 ± 7.6	*<0.001*
MIDAS	79 ± 40.6	27.5 ± 27.8	−51.9 ± 41.7	*<0.001*	52.7 ± 26.1	18.4 ± 19.9	−34.3 ± 28.1	*0.018*	88.3 ± 41.1	28.7 ± 28.4	−62.3 ± 40.5	*<0.001*
MIBS-4	10 ± 2.6	5.5 ± 2.4	−4.6 ± 2.3	*<0.001*	9.9 ± 2.1	5.0 ± 1.6	−4.8 ± 1.3	*<0.001*	10 ± 2.8	5.7 ± 2.6	−4.6 ± 2.6	*<0.001*

∆: difference between weeks 9–12 and baseline.

**Table 4:** Demographic and clinical features of patients with HFEM or CM who previously failed subcutaneous anti-CGRP mAbs treatment.

	All Patients	HFEM	CM
**Patients**	10	3	7
**Age**, *years*	49.8 ± 11.3	54 ± 16.5	48 ± 9.4
**Females**	7 (70.0%)	2 (66.7%)	5 (71.4%)
**BMI**	23.3 ± 2.2	23.5 ± 1.9	23 ± 2.4
**Age at onset**, *years*	14.7 ± 5.9	14.7 ± 5	14.7 ± 6.7
**MMD**	12.7 ± 1.5	12.7 ± 1.5	-
**MHD**	26.9 ± 3.8	-	26.9 ± 3.8
**NRS score**	8.2 ± 0.9	8.3 ± 0.6	8.1 ± 5.2
**MO**	7 (70%)	-	7 (100%)
**MO duration**	8.1 ± 5.2	-	8.1 ± 5.2
**Unilateral pain**	8 (80%)	2 (66.7%)	6 (85.7%)
**Unilateral cranial autonomic symptoms**	2 (20%)	1 (33.3%)	1 (14.3%)
**CAPS**	0.9 ± 1.7	0.7 ± 1.1	1 ± 1.9
**Allodynia**	4 (40%)	1 (33.3%)	3 (42.8%)
**ASC-12**	1.5 ± 2.9	0	2.1 ± 3.4
*0–2*	8 (80%)	3 (100%)	5 (71.4%)
*3–5*	0	0	0
*6–8*	2 (20%)	0	2 (28.6%)
**Dopaminergic symptoms**	4 (40%)	0	4 (57.1%)
**Monthly analgesic intake**	41.4 ± 60.7	10.7 ± 3.1	54.6 ± 69.7
**Triptan responders** *	7 (100%)	2 (100%)	5 (100%)
**Pts using concomitant prophylaxis**	3 (30%)	0	3 (42.8%)
**Prior treatment failures**	4.9 ± 2	3.7 ± 0.6	5.4 ± 2.2
*2–4*	5 (50%)	3	2 (28.6%)
*>4*	5 (50%)	-	5 (71.4%)
**Onabotulinum toxin A responders** **	0 (0/7%)	0 (0/2%)	0 (0/5%)
**Pts with ≥1 comorbidity**	4 (40%)	1 (33.3%)	3 (42.8%)
**Pts with psychiatric comorbidities**	2 (20%)	0	2 (28.6%)
**HIT-6 score**	67.7 ± 4.9	65.7 ± 0.6	68.6 ± 5.8
**MIDAS score**	77.8 ± 51.2	58.7 ± 41.7	86 ± 55.6
**MIBS-4**	10.3 ± 3.5	10.3 ± 2.1	10.3 ± 4.1
**Eptinezumab dose**			
*100 mg*	10 (100%)	3 (100%)	7 (100%)

Data are reported as or mean ± SD or proportion (%). HFEM: High frequency episodic migraine, CM: Chronic migraine, BMI: Body mass index, MMD: Monthly migraine days, MHD: Monthly headache days, NRS: Numerical rating scale, MO: Medication overuse, CAPS: cranial autonomic parasympathetic symptoms scale, ASC-12: allodynia symptom checklist, HIT-6: Headache Impact Test-6, MIDAS: Migraine Disability Assessment Scale, MIBS-4: Migraine Interictal Burden Scale. * Proportion calculated on the subjects who were treated with triptans, ** Proportion calculated on the subjects who were treated with onabotulinum toxin A.

**Table 5:** Change in monthly migraine days (MMD), monthly headache days (MHD), monthly analgesic intake (MAI), Numerical Rating Scale (NRS) score, Headache Impact Test-6 (HIT-6) score, Migraine Disability Assessment Scale (MIDAS) score and Migraine Interictal Burden Scale (MIBS-4) score from baseline to weeks 9–12 in patients with HFEM or CM who previously failed subcutaneous anti-CGRP mAbs treatments.

	Baseline	Weeks 9–12	∆	*p*
MMD *	12.7 ± 1.5	11 ± 5	−1.7 ± 4.1	*0.560*
MHD **	26.9 ± 3.8	9.4 ± 9.4	−17.4 ± 10.3	*0.004*
MAI	41.4 ± 60.7	9.4 ± 6	−32 ± 61.3	*0.133*
NRS	8.2 ± 0.9	4.9 ± 1.5	−3.3 ± 1.6	*<0.001*
HIT-6	67.7 ± 4.9	58.3 ± 9.3	−9.4 ± 8.9	*0.009*
MIDAS	77.8 ± 51.2	21.3 ± 29.3	−56.5 ± 48	*0.005*
MIBS-4	10.3 ± 3.5	5.8 ± 3.3	−4.5 ± 2.5	*<0.001*

∆: difference between weeks 9–12 and baseline. * MMD: data refer to the 3 patients with HFEM ** MHD: data refer to the 7 patients with CM

## Data Availability

The patients’ data are available at our outpatient department (IRCCS San Raffaele, Roma, Italy).
